# Multiple attention-based encoder–decoder networks for gas meter character recognition

**DOI:** 10.1038/s41598-022-14434-0

**Published:** 2022-06-20

**Authors:** Weidong Li, Shuai Wang, Inam Ullah, Xuehai Zhang, Jinlong Duan

**Affiliations:** 1 Key Laboratory of Grain Information Processing and Control, Henan University of Technology, Ministry of Education, Zhengzhou, 450001 China; 2 College of Information Science and Engineering, Henan University of Technology, Zhengzhou, 450001 China

**Keywords:** Computer science, Mathematics and computing

## Abstract

Factories swiftly and precisely grasp the real-time data of the production instrumentation, which is the foundation for the development and progress of industrial intelligence in industrial production. Weather, light, angle, and other unknown circumstances, on the other hand, impair the image quality of meter dials in natural environments, resulting in poor dial image quality. The remote meter reading system has trouble recognizing dial pictures in extreme settings, challenging it to meet industrial production demands. This paper provides multiple attention and encoder–decoder-based gas meter recognition networks (MAEDR) for this problem. First, from the acquired dial photos, the dial images with extreme conditions such as overexposure, artifacts, blurring, incomplete display of characters, and occlusion are chosen to generate the gas meter dataset. Then, a new character recognition network is proposed utilizing multiple attention and an encoder–decoder structure. Convolutional neural networks (CNN) extract visual features from dial images, encode visual features employing multi-head self-attention and position information, and facilitate feature alignment using the connectionist temporal classification (CTC) method. A novel two-step attention decoder is presented to improve the accuracy of recognition results. convolutional block attention module (CBAM) reweights the visual features from the CNN and the semantic features computed by the encoder to improve model performance; long short-term memory attention (LSTM attention) focuses on the relationship between feature sequences. According to experimental data, our system can effectively and efficiently identify industrial gas meter picture digits with 91.1% identification accuracy, faster inference speed, and higher accuracy than standard algorithms. The accuracy and practicality of the recognition can fulfill the needs of instrument data detection and recognition in industrial production, and it has a wide range of applications.

## Introduction

Gas meters for industrial applications are broadly employed in national defense, transportation, gas, electric generation, and other industries^1^. In recent years, the usage of meters in industrial production has increased dramatically due to the increased manufacturing scale. Due to their ease of installation and extended life cycle, mechanical gas meters are widely used in gas plants, water plants, substations, and other fields. However, the existing remote meter reading system has partially solved the problems of long cycle time and high cost of manual meter reading^2^. It still cannot accurately and quickly identify dial characters in extreme imaging environments, which prevents factories from accurately grasping real-time data in industrial production and impedes the development and progress of industrial intelligence. Recognizing digital characters in meter images is crucial stage in industrial meters’ remote meter reading system. Researchers at home and abroad are presently exploring the application of meter character recognition using various strategies to increase the accuracy of gas meter character identification^3–5^. Although these algorithms can automatically yield more accurate instrument recognition results under ideal conditions, the process is time-consuming. The operating procedure is inefficient and difficult to promote, mainly because of the weak recognition effect under extreme environmental circumstances. Using the character recognition based on the template matching method as an example^6^, it is necessary to manually create a standard template identification library with the exact size specifications and then adjust the image size of the characters to be recognized to match the image size of the standard template library. After segmentation, calculate the similarity between each character to be recognized and all characters in the template library, get the recognition result based on the similarity size, and output the result. Although this method is inexpensive, it necessitates a significant amount of preceding work, the computation is time-consuming, and different dials are required to create different character templates. Furthermore, the approach works well with meters with good imaging quality and standardized characters. Its identification performance for dials with unusual characters is low, and its imaging quality in meter images is poor. In recent years, due to the ability of computer vision methods based on deep learning to eliminate inconsistencies and errors in the results, a large number of computer vision methods have replaced traditional manual operations are widely used in various aspects^7^, such as automatic identification systems^8^, defect detection^9^, etc. For example, Litman et al.^10^ proposed a stacked architecture for the encoder–decoder character recognition method. This method employs Bi-directional Long Short-Term Memory (BiLSTM) as a stacking structure for the first time to compute contextual features repeatedly; nevertheless, the stacking block is up to 12 layers thick, resulting in considerable computational overhead and sluggish inference. Additionally, BiLSTM is only effective across a limited range of extensive distances.

Observation is the source of inspiration, and the attention mechanism is crucial in human vision. Character recognition has been successfully applied using attention techniques^11,12^. A novel encoder–decoder architecture based on a multi-attention mechanism is proposed in this study. Unlike^10^, our encoder comprises three encoder blocks containing a multi-head self-attention layer and a feedforward neural network layer. A novel two-step attention mechanism, incorporating CBAM attention^13^ and LSTM attention^14^, constructs the decoder. The CTC method is also used to increase the accuracy of gas meter character recognition by assisting with feature alignment. As a result, this research combines several attentions in a novel way to avoid the loss of relevant visual and semantic characteristics while improving the accuracy of dial character identification under harsh settings; simultaneously, it parallelizes the calculation to speed up inference. The main contributions of this paper are summarized as follows.

- The suggested method employs computer vision and deep learning to identify gas meter characters under harsh imaging conditions. It helps to advance the use of deep learning in manufacturing.
- A unique character recognition model is proposed based on a multi-attention and encoder–decoder architecture. The encoder uses a multi-head self-attention mechanism to compute the image’s semantic information, which it then merges with visual features to increase character identification accuracy. Meanwhile, the CTC method helps to align features and speed up model convergence.
- We present a novel two-step attention decoder. The initial step is CBAM attention, followed by LSTM attention. The two-step attention decoder can simultaneously pay attention to spatial, channel, and temporal multidimensional information.
- Experimental results on gas meter datasets under difficult environmental circumstances and public datasets, reveal that our technique delivers state-of-the-art character recognition performance. Its accuracy and practicality make it suitable for detecting and recognizing meter data in industrial operations.

## Related works

The most critical step in the instrument recognition process is obtaining precise and trustworthy character recognition results. Earlier studies^15–17^ focused on individual character studies, acquiring meter dial images, segmenting them into individual characters, performing recognition, and eventually aggregating the recognition results into sequences as the final result, such as the template matching method employed in^6^. Zhang et al.^18^ concentrated on the structural characteristics of individual characters and described the critical strategies for character recognition based on structural characteristics. The structural feature technique identifies characters primarily based on their structural traits. To start with, the characters to be recognized must have distinct structural characteristics. The greater the magnitude of such differences, the better; next, character comparison is performed using the extracted differentiated features to generate character recognition results. However, during the actual acquisition of gas meter dial images, various phenomena such as insufficient character differences, too much character data, and character ghosting occur, resulting in a substantial reduction in the method’s recognition accuracy. According to Jaderberg et al.^19^, the crucial issue of individual character identification is obtaining the appropriate character cut-off region, cut-off recognition, and the accurate aggregation of characters into sequences. Lee et al.^20^ used a sliding window approach in the character cutout candidate box, while Bai et al.^21^ used a linked region method. These two strategies are simple to implement and are often employed by researchers. However, these two approaches are only appropriate for characters with regular regions and considerable chromaticity deviations from the character backdrop. Identifying cut-off region candidate boxes might be challenging in some complex cases, such as characters with similar backdrop colors and fuzzy characters.

Deep learning is a multi-level representation learning method in which simple but nonlinear modules are combined to change a representation at one level (beginning from the original input) into a higher-level abstract representation^22^. Deep learning-based character recognition is a fundamental and challenging problem in computer vision, and it is widely utilized in business offices^23,24^, finance^25,26^, medicine^27,28^, automotive^29,30^, and industrial^31,32^. The rich knowledge of context^33^ and structured prediction algorithms^34^ are combined in these systems to make significant improvements. Shi et al.^35^ suggested a CRNN network that uses a canonical convolutional neural network to extract input picture features. The technique employs BiLSTM^36^ to model the retrieved picture characteristics in time sequence, then uses the CTC algorithm^37^ to decode the best character sequence, avoiding the need to segment the characters and normalize the character scaling. It can quickly process sequences of any length but it has the drawbacks of poor training convergence and erroneous recognition of letters with significant angular tilt. A scene text recognition model based on a visual attention mechanism was proposed by Wojna et al.^38^. The model initially utilizes CNN to extract image features and then uses LSTM to model the extracted image characteristics in time series to generate temporal features, with the attention model serving as a decoder. This technique outperforms prior best models on the FSNS dataset, with a correct rate of approximately 85%, but it is less robust and prone to noise. Yu et al.^39^ suggested an SRN model based on ResNet50^40^ and transformer unit as the backbone network to extract the visual information from the input image. Then, a global semantic inference module was implemented to extract the semantic information from the input image, which combined the semantic and visual data to decode the character sequence. On the other hand, the model used a serial decoder, which produced a prediction efficiency that was far too low. Yang et al.^41^ introduced the HGA-STR model based on the transformer structure and used CNN to extract features, keeping the output 2-dimensional features and decoding with a bidirectional decoder. Noise is added in the feature extraction stage, despite the enhanced performance. Character recognition has also begun to use segmentation-based algorithms^42,43^ and target detection methods^44–47^.

In contrast to the previous models, Litman et al.^10^ proposed a stacked architecture encoder–decoder character recognition method that employs a stacked block architecture to train deep BiLSTM encoders, resulting in improved context-dependent coding and state-of-the-art performance in publicly available data sets. However, the approach has a significant computing overhead and slow inference, making it unsuitable for character recognition of characters captured under harsh conditions. Inspired by this, we present a novel encoder–decoder model based on the attention mechanism that focuses on the picture’s shallow visual aspects and deep semantic information, conserving more image details while boosting inference speed.

## Method

### System overview

**Figure 1:**
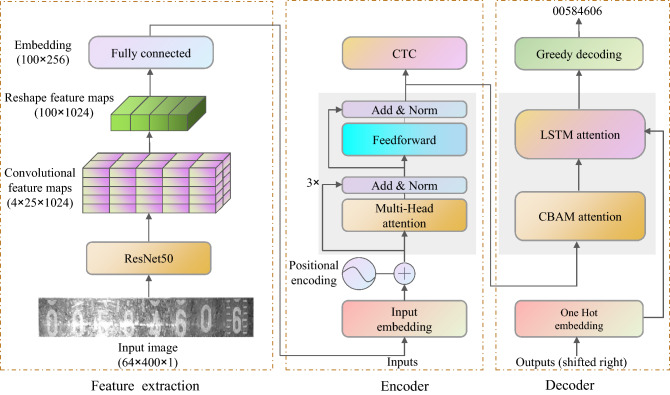
Model of our proposed recognition system.

As illustrated in Fig. 1, our suggested model comprises three phases: feature extraction, encoder, and decoder. The backbone network for the feature extraction phase is pre-trained ResNet50. Conv1, conv2_x, conv3_x, conv4_x, and conv5_x are the five modules that make up ResNet50. We use the first four portions of ResNet50 to extract features of acceptable size: conv1, conv2_x, conv3_x, and conv4_x.

The model is fed a 644001 (HWC) grayscale image, and the features are extracted using a pre-trained ResNet50 to produce a 4251024 (HWC) feature map. The feature map is next reshaped and dimensionally altered to produce a feature map with a size of 1001024 (HWC). Meanwhile, a full connection to the feature map is established to finish the feature map’s embedding. The embedding vector is supplied to the model’s encoder, along with the position encoding, and the encoded output is acquired after three encoders. The decoder employs a new two-step attention decoder to decode the visual data derived from the CNN, the semantic information computed in the encoder. It can also concentrate on the internal links between feature sequences.

The inference flow of the model is as follows.

1. Preprocessing: The input image is resized and converted to 64400 (HW) to a grayscale map in the pre-processing stage.
2. Feature Extraction: Image features are extracted using pre-trained ResNet50 during the feature extraction stage.
3. Encoder: The Embedding feature vector is encoded many times during the encoding phase, and the semantic features are extracted via a multi-headed self-attentive technique.
4. Decoder: A novel two-step attention mechanism decoder is used in the decoder stage.

### Preprocessing stage

**Figure 2:**
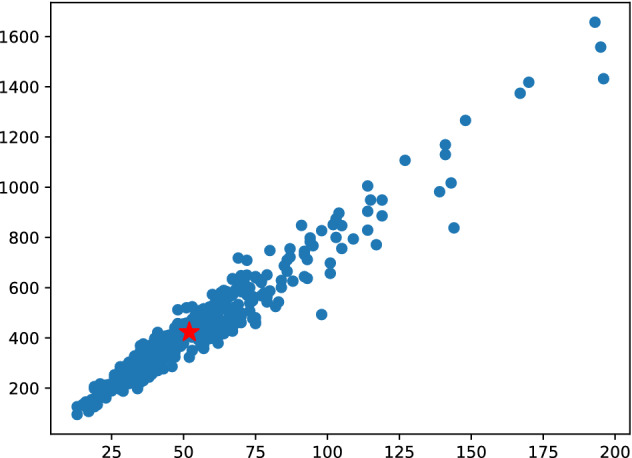
K-means clustering results for the gas meter dataset.

The images acquired by the remote meter reading device are of various sizes; thus, we utilize K-means clustering to determine the most aggregated image size and exhibit the clustering results in Fig. 2. The cluster center is chosen as the image’s fixed size during preprocessing, and the cluster center is around 64,400 (HW); therefore, the image is uniformly adjusted to this size.

### Feature extraction stage

The feature extraction process extracts visual information from the input image using a convolutional neural network (CNN). By combining accuracy and speed factors, the pre-trained ResNet50 is chosen as the backbone network for image feature extraction in this article. The output of the feature extraction step is 256 channels by N columns. Specifically, the feature extractor gets an input image and outputs a feature map $F=[f_1, f_2,\dots , f_N]$. The attention map is generated after feature extraction using a fully connected layer. The visual features are then obtained by multiplying the feature vectors by the attention features using the mathematical formula $V = [v_1, v_2,\dots , v_N]$, where each column can represent a frame of the visual feature.

### Encoder process

Instead of using a typical recurrent neural network (RNN), the model’s encoder employs a multi-head self-attention layer, which can be computed in parallel, overcoming the problem that RNN networks cannot be parallelized and to save training and inference time. We then go through numerous aspects of the encoder phase in further detail.

- Input Embedding.
  Features must be embedded before they can be entered into Encoder. The purpose of feature embedding is to reduce the computing effort by downscaling the features to the required size. This experiment uses a fully-connected layer to map a feature vector size 1024 to 256. This can minimize the features’ dimensionality and speed up model training and inference.
- Positional Encoding.
  To overcome the problem of the network being unable to model the input features as time series, resulting in the model being unable to understand the features’ backward and forward sequential relationships. To discover the time-series link of the projected results, we must provide location information to the model in a certain way. Because the location encoding and the feature vector must be summed, the dimensionality of the position encoding and the feature vector are the same. A learnable position code and a position code constructed using sine and cosine functions of different frequencies are the two methods for constructing a position code. In this work, we employ the second, and the formulas are as follows:
  1 $$\begin{aligned} \begin{array}{l} P E_{(p o s, 2 i)}=\sin \left( p o s / 10000^{2 i / d \,{\text{model}}}\right) \\ P E_{(p o s, 2 i+1)}=\cos \left( p o s / 10000^{2 i / d \,{\text{model}}}\right) \end{array} \end{aligned}$$
  where *pos* is the character’s location in the string, *i* is the index of the feature vector dimension, and the $d_{model}$ is the position encoding dimension. By using above mentioned formulas, the following are some of the advantages.
  Each position has a unique position code.
  Suppose the maximum length feature in the current dataset is 10, and a feature of length 12 needs to be processed. In that case, the formula can be used to generate the last two position codes immediately, thus improving the fault tolerance and robustness of the model.
  The relative position relationship between features can be easily determined during training and inference. The trigonometric function of $PE_{pos+k}$ can be used to express information at any location *pos*, and the formulas are shown below:
    2 $$\begin{aligned} \begin{array}{c} \cos (\alpha +\beta )=\cos (\alpha ) \cos (\beta )-\sin (\alpha ) \sin (\beta ) \\ \sin (\alpha +\beta )=\sin (\alpha ) \cos (\beta )+\cos (\alpha ) \sin (\beta ) \end{array} \end{aligned}$$
    Each feature position must have its position encoding in the form of a sine wave. The time and phase of the sine wave vary depending on the dimension of the feature. The letters *pos* and *i* represent the feature vector’s position and dimension. The sine and cosine functions of different frequencies are used to create a position code the same size as the feature vector, which is then superimposed on the feature vector and fed into the encoder, and the formula is as follows:
    3 $$\begin{aligned} X_{\text{embedding }}=X_{\text{embedding }}+X_{\text{pos }} \end{aligned}$$
- Multi-Head Self-attention.
  Three matrices, *Query*, *Key*, and *Value*, are used as inputs to the multi-head attention mechanism. The three matrices are fed into the fully connected layer, which then performs a scaled dot-product attention operation. The process needs to be computed h times, the h-head attention mechanism. The weights of the input feature vectors determine the model’s attention, known as the self-attention mechanism, which allows the model to obtain correlations between the feature vectors and so preferentially focuses on the characteristics of interest. *Query*, *Key*, and *Value* (abbreviated as *Q*, *K*, and *V*) matrices, which represent query, key, and value, are the primary sources of attention. They have the same dimensions as the feature vector. *Query*, *Key*, and *Value* are obtained by multiplying the feature vector $X_{embedding}$ by the three weight matrices $W_Q$, $W_K$, and $W_V$, respectively, and the computation procedure is given in (4).
  4 $$\begin{aligned} \begin{array}{l} Q=X_{\text{embedding }} * W_{Q} \\ K=X_{\text{embedding }} * W_{K} \\ V=X_{\text{embedding }} * W_{V} \end{array} \end{aligned}$$
  The multi-head self-attention mechanism separates the feature vector $X_{embedding}$ into multiple parts equally, with each head representing one of the features. It should be noted that the number of heads must be divisible by the feature vector dimension. Assume the number of heads is *h* and the eigenvector dimension is $d_{model}$. Following multiple heads, *Q*, *K*, and *V* are trimmed into single-headed matrices, and their last dimension $d_k$ is determined using Eq. (5).
  5 $$\begin{aligned} d_{k}=d_{\text{model }} / h \end{aligned}$$
  After multiplying *Q* and *K*, we get the $QK^T$ matrix, dividing by $\sqrt{d_{k}}$. The method of scaling dot product attention is the name of the process. Its function is to prevent the value after multiplication from becoming excessively large and act as a normalizer. All data are mapped to the (0, 1) interval by the softmax activation function, resulting in a correlation of 1 between each feature and the other characteristics. The attention matrix is then obtained by multiplying by the *V* matrix. The computational formula is given by (6).
  6 $$\begin{aligned} {\text{Attention}}(Q, K, V)={\text{softmax}} \left( \frac{Q K^{T}}{\sqrt{d_{k}}}\right) V \end{aligned}$$
- Add & Norm.
  The *Add* is a residual operation. The specific operation is to add the preceding layer’s input *x* and the output *SubLayer*(*x*) to get $x+SubLayer(x)$. The addition of the residual operation is intended to mitigate the drawbacks of network degradation by learning the residuals. The attention module uses the formula (7) to calculate residuals.
  7 $$\begin{aligned} X_{\text{embedding}}+{\text{Attention}}(Q, K, V) \end{aligned}$$
  The encoder uses the *LayerNormalization* to alleviate the skew variable shift phenomenon, minimize computation, and improve data variability. On each dimension of the feature vector, the *LayerNormalization* executes a normalization operation. Furthermore, the feature vector conforms to the conventional normal distribution because the normalized expectation ${\mu }$ is 0, and the standard deviation ${\sigma }$ is 1.
- Feedforward.
  The feedforward neural network enhances the classification ability of the model. The network goes through two layers of a simple linear mapping $W_1$, which is then fed into a relu activation function with the formula shown in (8).
  8 $$\begin{aligned} X_{\text{hidden}}+{\text{Relu}} \left( X_{\text{hidden}} * W_{1} * W_{1}\right) \end{aligned}$$
  The above five components constitute the Encoder module of the model.

### Decoder process

**Figure 3:**
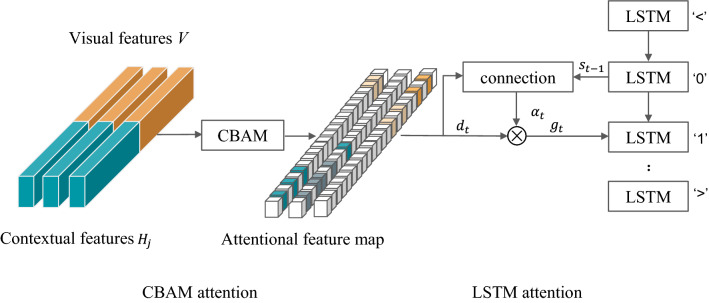
Architecture diagram of our two-step attention decoder.

Figure 3 depicts our model’s utilization of a novel two-step attention mechanism decoder. The first phase is CBAM, a lightweight attention module that computes attention in the feature channel and spatial dimensions. The channel attention submodule and the spatial attention submodule determine the weights, reweighted to yield the attention features $D^{\prime }$.

The channel attention submodule feds the feature map *F* of HWC and uses a global average pooling and maximum pooling, respectively, to generate two 11C feature maps based on the height H and width W. The multilayer perceptron (MLP) then adds the two feature vectors element by element, and the weight coefficients $W_C$ are produced using the sigmoid activation function. Finally, the original feature map *F* is multiplied by $W_C$ weight coefficients. Adaptive feature refinement is used to create the new attentional feature map *D*. The equation is shown in (9).

9 $$\begin{aligned} D=F \times W_{C} \end{aligned}$$

The spatial attention submodule receives the above-mentioned new feature map *D* as an input. After a global average pooling and maximum pooling, respectively, based on the latitude of the feature channels. We generate two corresponding feature maps of HW1; then, after stitching these two feature maps together, we obtain the weight coefficients $W_S$ using a convolution layer and a sigmoid activation function. Finally, we multiply the weight coefficients $W_S$ by the input feature map *D* to get the attention features $D^{\prime }$. The formula is shown in (10).

10 $$\begin{aligned} D^{\prime }=D \times W_{S} \end{aligned}$$

In the second step, the attention decoder decodes the attention feature $D^{\prime }$, and a character $y_t$ is decoded at each time step t. The number of time steps equals the length of the longest string in the dataset plus one. The formula is given in (11), wherein $\alpha _{t} \varepsilon R^{N}$, $\alpha _{t}$ represents the attention of the decoder at time t, and $R^{N}$ represents the attention of all time steps.

11 $$\begin{aligned} \begin{array}{l} e_{t, i}=w^{T} \tanh \left( W s_{t-1}+V d_{i}^{\prime }+b\right) \\ \alpha _{t, i}=\exp \left( e_{t, i}\right) / \sum _{i^{*}=1}^{n} e_{t, i^{*}} \end{array} \end{aligned}$$

where *b*, *w*, *W*, and *V* are trainable parameters, is the hidden state of the LSTM unit within the decoder at time t, and $d^{\prime }$ is a column of $D^{\prime }$. The decoder linearly combines the columns of $D^{\prime }$ into a vector *G*, which is called a glimpse:

12 $$\begin{aligned} g_{t}=\sum _{i=1}^{n} \alpha _{t, i} d_{i}^{\prime } \end{aligned}$$

Then, the input of one LSTM unit of the decoder is shown in Eq. (13), where $(g_t,y_{t-1})$ denotes the connection between the One Hot codes of $g_t$ and $y_{t-1}$.

13 $$\begin{aligned} \left( x_{t}, s_{t}\right) ={\text{LSTMCell}}\left( s_{t-1},\left( g_{t}, f\left( y_{t-1}\right) \right) \right) \end{aligned}$$

Finally, for each computational time step t, the probability $p(y_t)$ of the character is as follows:

14 $$\begin{aligned} p\left( y_{t}\right) ={\text{soft}} \max \left( W_{o} x_{t}+b_{o}\right) \end{aligned}$$

After decoding the above steps, the probability distribution of the characters can be obtained, and then the recognition results can be obtained.

### Loss function

We need to avoid the problem of neighboring areas being recognized as the same character when recognizing input image features in regions due to variables such as character distortion. Therefore the CTC algorithm is employed in our model to align the input features to the data labels, and the features aligned by the CTC algorithm are used as the decoder’s input. The experiment used the CTC technique for additional training, which is the same as utilizing two loss functions: the CTC loss on the encoder side and the cross-entropy loss on the decoder side. The CTC loss accounts for ${\gamma }$ times of the total loss, while the cross-entropy loss occupies $1-{\gamma }$ times. The formula for the loss function is followed as:

15 $$\begin{aligned} L_{\text{total }}=\gamma L_{C T C}+(1-\gamma ) L_{C E} \end{aligned}$$

## Results and discussion

### Datasets

The dataset utilized was obtained from photographs acquired by a remote meter reading device. It included a variety of scenes such as day, night, sunny day, lighting, different angles, etc. We chose 30,000-m photos with poor imaging conditions like dim light, artifacts, heavy shadows, blurring, reflections, etc. After completing hand annotation, we saved the results in an XML tag file, with each XML matching one image in the dataset. To improve the generalization ability and robustness of the model, thus making the model provides more accurate and reliable predictions. This paper uses data enhancement techniques such as salt & pepper noise, speckle noise, Gaussian noise, motion blur, random wipe, random crop, random flip, and random rotation to expand the dataset to 70,000. Then the dataset is randomly divided into 7:3:1 as the training set, validation set, and testing set, and each part contains corresponding labels.

The publicly available datasets **IIIT5k**^48^, **SVT**^49^, and **ICDAR 2013**^50^ are also used in this paper to demonstrate further that our strategy is viable and successful. **IIIT5k** consists of 2000 training and 3000 testing images cropped from Google image searches. **SVT** is a dataset collected from Google Street View images and contains 257 training, and 647 testing word-box cropped images. **ICDAR 2013** contains 848 training and 1015 testing word-box cropped images.

### Implementation details

The experimental environment is Ubuntu 20.04 operating system, Intel®  Core$^{\mathrm{TM}}$ i9-9900K processor, 32 GB RAM, RTX 2080Ti*2 GPU, deep learning framework using Pytorch1.8, and general-purpose parallel computing architecture CUDA11.1.

We used the pre-trained ResNet50 from ImageNet^51^ as the backbone network in our studies. ResNet50 contains conv1, conv2_x, conv3_x, conv4_x, conv5_x. The first four components of ResNet50 were used: conv1, conv2_x, conv3_x, conv4_x. The images are uniformly processed into 64400 (HW) single-channel grayscale images during training and inference. The Adam optimizer is used to train the model in this paper. To regulate the weight distribution of momentum and current gradient, we set the exponential decay rate $\beta _1$ = 0.9; to control the contribution of the prior gradient squared, we set the exponential decay rate $\beta _2$ = 0.98. The training batch size is 64, and after 15,000 iterations, the training is completed, and the results are given.

The model outputs the conditional probability distribution of characters during the training inference phase. In this paper, we employ greedy search to decode characters. At each time step, we select the character with the highest probability and then intercept the string between the start and end characters as the character content of the image recognized by the model.

### Analysis of MAEDR model and MAEDR + CTC experimental results

**Figure 4:**
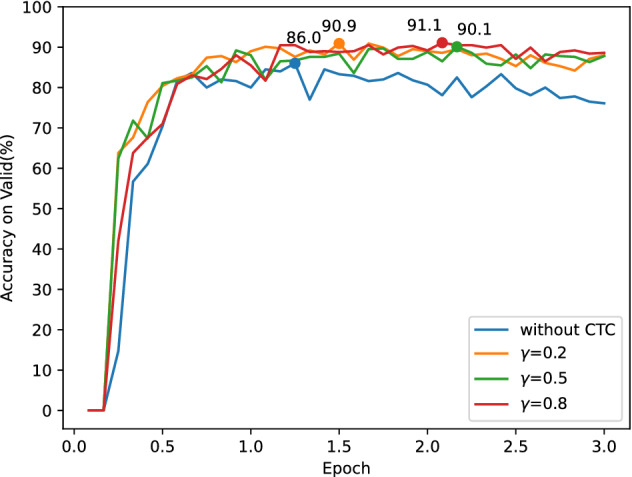
Performance comparison of MAEDR model (Ours-1) and MADER + CTC model (Ours-2).

The CTC algorithm is the primary variable in this section. The MAEDR model is combined with the CTC algorithm. The performance of the MAEDR model alone (Ours-1) and MADER + CTC (Ours-2) is next investigated. Figure 4 shows the accuracy of the test set when the CTC loss ${\gamma }$ is equal to 0.2, 0.5, and 0.8.

As shown in Fig. 4, the accuracy of the MADER+CTC model (Ours-2) has significantly improved. The CTC algorithm aids the model in aligning the data labels, which boosts the model’s accuracy. The MAEDR model alone (Ours-1) obtains a maximum accuracy of 86.0%. However, the Ours-2 recognition accuracy outperforms the MAEDR model alone for ${\gamma }$ equal to 0.2, 0.5, and **0.8**, respectively, with 90.9%, 90.1%, and **91.1%** accuracy. Therefore, we choose CTC loss ${\gamma }$ = 0.8.

### Evaluation of character recognition

We did numerous trials on the gas meter data set and performed the experimental analysis in this section to evaluate the suggested algorithm’s validity, accuracy, and feasibility. As previously stated, the method’s accuracy and efficiency in harsh settings are critical characteristics. As a result, we use two quantitative criteria to evaluate the model’s performance: accuracy and inference time. We compare the recognition results with those of several advanced character recognition methods: SCATTER^10^, CRNN^35^, SRN^39^, and HGA-STR^41^.

**Table 1:** Recognition performances of our method compared with other baselines on the gas meter dataset.

Model	Accuracy (%)	FPS	Parameter number
CRNN	87.5	142	**8317963**
SCATTER	86.2	41	119375120
SRN	87.4	71	57199301
HGA-STR	83.4	17	33916725
MAEDR (Ours-1)	86.0	**200**	31437218
MAEDR + CTC (Ours-2)	**91.1**	**200**	31437218

In each column, the best performing result is shown in bold, and the second best result is shown in underline.

Table 1 shows the recognition results of several algorithms for the gas meter dataset under harsh image conditions. Our method exceeds other well-known techniques, not only in terms of accuracy but also in terms of inference speed. Both based on the encoder–decoder framework, SCATTER^10^, and HGA-STR^41^, perform poorly in accuracy and inference time. SCATTER uses a stacked repetitive processing architecture to increase the computational effort in extracting features, and the decoder is a selective decoder with low parallelism. The HGA-STR model extracts feature using CNN and preserve the output features as two-dimensional. The model employs pooling operations to acquire global information to generate one-dimensional vectors. The decoding technique is the same as the transformer structure, and the model uses two directions for decoding at more incredible speed. However, the global information obtained by the encoder introduces noise when the low-level features are gradually merged in the decoder. Unlike the encoder–decoder structure described above, our technique encodes convolutional features using a multi-head self-attention encoder. The encoder output’s high-level attention information lessens the effect of noise. Meanwhile, the CTC algorithm is used to solve the alignment of input features and output labels, which further reduces the impact of noise. Our innovative decoder is split into two halves. The first portion, which focuses on feature channels and spatial dimensions, employs a convolutional attention module (CBAM). The second half uses an LSTM attention module that focuses on the feature sequence’s associations. Based on the height and width of the feature map, the channel attention module considers a global average pooling and maximum pooling, respectively. This process is used to aggregate the spatial information of the feature maps, compress the spatial dimensions of the feature maps in a shared network, and sum the elements one by one. The spatial attention submodule compresses the channels in the feature channel dimension by using a global average pooling and maximum pooling. This process improves the overall recognition accuracy of the model by suppressing noise in the boundary and background information while preserving visual features and semantic information. The CBMA module does not significantly increase the number of parameters in the network. At the same time, it can use computing resources to improve the model effect by enhancing critical aspects and suppressing minor features. After getting the feature attention sequence, the LSTM module focuses on the sequence’s internal relationship and decodes the final string.

Our method likewise performs well compared to the non-encoder–decoder structures of the CRNN^35^ and SRN^39^ techniques. Even though the CRNN model can handle sequences of any length without character segmentation, scaling, or normalization, it transcribes the final result using the CTC algorithm, which inaccurately identifies characters with large angular tilt occlusion anomalies. Furthermore, the SRN model uses serial decoding, which leads to too low prediction efficiency.

To encode visual features, we employ multi-headed self-attention and location information encoding. It uses a CTC algorithm to tackle the alignment problem between input features and output labels and a two-step attention decoder that includes a CBAM module and an LSTM attention module. Our MAEDR model performs well in extreme conditions such as image overexposure, artifacts, blur, missing character display, and occlusion and has the most remarkable performance in two crucial metrics: accuracy and inference time. To assess the performance of the novel algorithm proposed in this paper on a dataset with poor imaging quality and to justify the proposed novel decoder, we created a dataset with even poorer imaging quality using data enhancement techniques such as adding noise, fogging, etc. and ran many more experiments with the best-performing CRNN model mentioned above. The results are shown in Table 2.

**Table 2:**
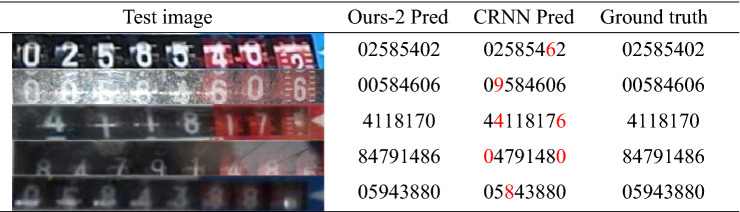
Recognition performances of our method compared with CRNN on the gas meter dataset.

In terms of recognition accuracy and speed, our MAEDR + CTC (Ours-2) model surpasses the prior model after analysis. The MAEDR + CTC (Ours-2) model can recognize character content better in extreme circumstances, with superior robustness, generalization ability, and significant application value.

### Experimentation on public datasets

In addition to the gas meter table image dataset under extreme conditions, we also conducted experiments on publicly available datasets IIIT5k^48^, SVT^49^, and ICDAR2013^50^ to further validate that our method is feasible and effective. We ran numerous experiments with earlier state-of-the-art algorithms on the public datasets: SCATTER^10^, CRNN^35^, SRN^39^, RobustScanner^52^, ABINet-LV^53^, and PREN2D^54^.

**Table 3:** Recognition performances of our method compared with SOTA on public datasets.

Model	Test datasets & accuracy(%)		
	IIIT5K	SVT	IC13
SCATTER	93.7	92.7	93.9
CRNN	82.9	81.6	89.2
SRN	94.8	91.5	95.5
RobustScanner	95.3	88.1	94.8
ABINet-LV	96.2	93.5	**97.4**
PRENatal2D	95.6	94.0	96.4
MAEDR (Ours-1)	95.1	**94.1**	95.2
MAEDR + CTC (Ours-2)	**96.3**	**94.8**	96.9

In each column, the best performing result is shown in bold, and the second best result is shown in underline.

Table 3 demonstrates that our method achieves surprising performance on the SVT dataset collected from Google Street View. It contains a large amount of data under extreme conditions such as occlusion, reflection, and dark light. Our approach achieves the competition on the SVT dataset thanks to a novel two-step attention decoder that focuses on the channel and spatial information of the images and the links within the feature sequences. Notably, our technique achieves equivalent SOTA performance on the IIIT5K and ICDAR2013 regular text datasets.

In summary, our model achieves the best recognition performance on two of the three baselines and the second-best performance on the other baseline. The most significant difference with the other methods is that our method performs better on data with extreme conditions.

### Ablation experiments

Furthermore, we conducted ablation studies on the CTC and CBAM modules to assess our proposed technique’s performance, particularly the two modules that make up the decoder (i.e., CBAM and LSTM). The ablation experiments for our suggested model MAEDR+CTC were effective, and the comparison findings are provided in Table 4. The results show improved accuracy in each example, indicating that each module is useful. The CTC algorithm, in particular, aids in the resolution of the correspondence between the input sequence and the label because character recognition requires not only the labeling of characters but also the labeling of their positions, which can be difficult in some cases. CTC-assisted training solves this alignment problem. CBAM denoise low-level features such as background texture feature at the early stage. They then gradually focus on the exact target, a high-level semantic. As a result, the module can pay more attention to images’ complex and detailed features and get better detection results. In other words, it proves that CBAM retains the essential information of images and suppresses the unnecessary ones, which results in a more discriminative feature for deep character recognition. Combining the two modules yielded the best results, demonstrating that our methodology is feasible and beneficial.

**Table 4:** Comparison of results of ablation experiments.

Methods	Test datasets & accuracy(%)			
	Our	IIIT5K	SVT	IC13
MAEDR+CTC	**91.1**	**96.3**	**94.8**	**96.9**
Without CTC	86.0	95.1	94.1	95.2
Without CBAM	85.4	95.6	93.2	95.7
Without CTC & CBAM	84.2	94.3	92.5	94.1

In each column, the best performing result is shown in bold.

## Conclusion

The poor recognition of dial pictures by remote meter reading systems in extreme instances is addressed in this research, which is challenging to meet industrial production needs. We offer a unique character recognition model based on a multi-attention mechanism and an encoder–decoder. Instead of using the usual RNN technique, the model’s encoder employs a multi-head self-attention mechanism, which allows for parallel computing. A novel two-step attention machine decoder is also proposed, obtaining the attention feature map following the first attention mechanism. The second attention mechanism concentrates on the temporal relationship between the features in the sequence, decoding the string in the image after multiple time steps. In addition, on the encoder side of the model, a CTC algorithm is added to aid in training. This technique is applied to handle the alignment problem between input features and output labels and achieves a **5.1%** improvement over the previous introduction. Experiments have shown that the model performs well in extreme scenarios such as overexposure, artifacts, blurring, missing character display, and picture occlusion, with an accuracy of **91.1%** on a gas meter dataset with poor imaging quality. The model’s accuracy and practicality make it suitable for instrument data detection and identification in industrial production, and it has a wide range of applications.

## Data Availability

The datasets generated and/or analysed during the current study are not publicly available due we are doing research on half-character recognition for our dataset but are available from the corresponding author on reasonable request.
